# Rheumatism—Its Nature, Its Pathology, and Its Successful Treatment

**Published:** 1886-11

**Authors:** 


RHEUMATISM: ITS NATURE, ITS PATHOLOGY AND ITS SUCCESSFUL TREATMENT. By T. J. Maclagan, M. D. New York: William Wood & Company, 56 and 58 Lafayette Place. 1886.

The practical bearing of the conception on the part of the author of this work, that rheumatism is allied to miasmatic disorders and to be met on general principles, does not quadrate with his view of the special adaptation of the salicyl compounds to its connection. It is evident that the fibrinous development associated with rheumatism is the prime element to be combated, and there is no remedy so potent in modifying this proclivity as calomel, which, according to the present writer's experience, is more uniformly successful in arresting its progress and preventing rheumatic endo-carditis than any other medicinal agent. That the alkaline treatment which is recommended in a class of cases in which the salicyl compounds fail to effect a cure, may aid in the elimination of the lactic acid, does not preclude measures for defibrination of the blood and modification of the hepatic function. The different forms of rheumatic manifestation receive full consideration, and the work is well worthy of the attention of practitioners, notwithstanding the salicylic thread which is woven into its fabric. This book is unique in presenting the author's name without surroundings of position, an innovation worthy of imitation by other writers.

